# A Meta-Analytical Investigation of the Gap between Measured and Predicted Inter-Population Genetic Diversity in Species of High Conservation Concern—The Case of the Critically Endangered European Mink *Mustela lutreola* L., 1761

**DOI:** 10.3390/genes12101555

**Published:** 2021-09-29

**Authors:** Jakub Skorupski, Johan Michaux, Przemysław Śmietana

**Affiliations:** 1 Institute of Marine and Environmental Sciences, University of Szczecin, Adama Mickiewicza 16 St., 70-383 Szczecin, Poland; przemyslaw.smietana@usz.edu.pl; 2 Polish Society for Conservation Genetics LUTREOLA, Maciejkowa 21 St., 71-784 Szczecin, Poland; 3 European Mink Centre, 5 Lipca 45 St., 70-374 Szczecin, Poland; 4 Laboratoire de Génétique de la Conservation, Institut de Botanique (Bat. 22), Université de Liège (Sart Tilman), Chemin de la Vallée 4, B4000 Liège, Belgium; johan.michaux@uliege.be; 5 Animal, Santé, Territoire, Risque Environnement-Unité Mixe de Recherche 117 (ASTRE), Université de Montpellier, Centre International de Recherche Agronomique pour le Développement (CIRAD), Institut National de la Recherche Agronomique, 34398 Montpellier, France

**Keywords:** conservation genetics, endangered species, European mink, meta-analysis, mtDNA, *Mustela lutreola*, population genetics, rarefaction, sampling completeness, sampling size

## Abstract

Although properly designed sampling in population genetic studies is of key importance for planning evidence-informed conservation measures, sampling strategies are rarely discussed. This is the case for the European mink *Mustela lutreola*, a critically endangered species. In order to address this problem, a meta-analysis aiming to examine the completeness of mtDNA haplotype sampling in recent studies of *M. lutreola* inter-population genetic diversity was conducted. The analysis was performed using the sample-size-based rarefaction and extrapolation sampling curve method for three populations—the Northeastern (Russia, Belarus and Estonia), the Western (France and Spain), and the Southeastern (Romania). The extrapolated values of the Shannon–Wiener index were determined, assuming full sample coverage. The gap between the measured and predicted inter-population genetic diversity was estimated, indicating that the identified level of sample coverage was the lowest for the NE population (87%), followed by the SE population (96%) and the W population (99%). A guide for sampling design and accounting for sampling uncertainty in future population genetic studies on European mink is provided. The relatively low sample coverage for the Russian population clearly indicates an urgent need to take conservation measures for European mink in this country.

## 1. Introduction

Sample size is a critical issue for measuring genetic variation, yet for population genetic studies, sampling strategies are rarely discussed [1,2,3]. Correct inference about intraspecific genetic variation requires the allele (or haplotype, as the equivalent of a combination of alleles) frequencies in a sample to accurately reflect their frequencies in the general population, and to capture real inter-population differences in genetic structure. The key parameters used in conservation genetics to measure genetic diversity are allelic (or haplotype) richness and expected heterozygosity [1,2,4,5,6]. An incorrect sampling strategy leads to ambiguous, inconclusive or false results for the abovementioned parameters, with erroneous data, which can have particularly damaging consequences when planning conservation measures [7,8,9].

Several strategies for determining the sample size appropriate for correctly estimating the true genetic diversity of wild populations have been proposed. An ex ante, empirical approach requires a pilot study and, therefore, previous knowledge of the genotype frequencies for a given population [4,10]. Another strategy is multiple sampling; however, in most cases, only a single sample from a population is taken, without accounting for sampling uncertainty [11]. Finally, sampling schemes can be validated ex post, by utilizing methods such as jackknifing [12], the Good–Turing frequency estimation [13], regression models [14], or rarefaction analysis [1], to account for unsampled (unmeasured) alleles or haplotypes [15]. Such approaches mainly aim to inform future sampling design [4].

Different authors have suggested different numbers of randomly sampled individuals—from four to six, in the case of high inter-population differentiation, to 20–30, for populations characterized by low or unknown levels of diversity or genetically depauperated, and even up to hundreds [1,8,16,17,18,19,20]. Generally, it is claimed that large sample sizes or a large number of variable sites analyzed increases the accuracy of the estimation of genetic diversity parameters, but the rate of increase is not linear [10,19,21]. A too-small sample size may result in significant errors in estimating the genetic diversity parameters, while a too-large sample size will inflate costs and research time [8,22,23].

One of the greatest limitations in research into the population genetics of threatened and endangered species is the often-very-limited access to a sufficient number of samples to capture their real genetic diversity [3]. This is the case for the European mink *Mustela lutreola*, a critically endangered species, with sparse, vanishing populations, often found in difficult-to-access areas, scattered across Russia, Romania, France and Spain [24]. The species has undergone a severe decline over the last 150 years due to habitat loss, extensive over-hunting and competition with the non-indigenous, invasive American mink *Neovison vison* [24,25]. It is categorized as critically endangered by the IUCN Red List of Threatened Species [24]. The sampling of individuals from rapidly declining populations is not only a logistic challenge, but also raises an ethical issue, so the optimization of sampling methods is of high importance for conservation.

Since it is postulated that research on critically endangered populations should include measures of genetic diversity, even if the sample size is not optimal [18], our goal was to examine if the sample sizes in recent population genetic studies of *M. lutreola* revealed the possible numbers of haplotypes (haplotype richness) present in the examined populations and to ex post quantify the potentially missing haplotypes in the samples, through a meta-analysis approach using the rarefaction method. By building on previous work, this study aimed not only to identify a possible gap between measured and predicted inter-population genetic diversity in preserved populations, but also to provide a guide for sampling design and accounting for sampling uncertainty in future population genetic studies on European mink.

## 2. Materials and Methods

In order to estimate the sampling completeness (the difference between the observed richness and the estimated asymptotic richness), a dataset consisting of results reported by Davison et al. [26], Michaux et al. [27,28], Korablev et al., [29] and Cabria et al., [30] was constructed (Table S1–S5). Such a meta-analytical approach allowed us to capture all the available data for the intraspecific genetic diversity of the species. To compare and unify the results obtained by different authors, the focus was on mitochondrial markers (mtDNA haplotypes), i.e., a 337-bp fragment of the *cytb* gene (GenBank acc. no. AF207720-AF207725) [26], and fragments of the control region of different sizes−617-730 bp (GenBank acc. no. AJ548803-AJ548820) [27,28], 525-526 bp (GenBank acc.no. JX982495-JX982502) [29] and 501-503 bp (GenBank acc. no. EU548035-EU548051) [30]. In total, 424 individuals were examined in the research mentioned.

The analyses were based on the number of haplotypes (haplotype richness) determined in these studies and assigned to three genetically distinguishable extant study populations (Table 1), i.e., the Northeastern (NE; inhabiting the Volga and the Dvina basin in Russia and, formerly, also the Vitebsk region in Belarus and Estonia), the Western (W; inhabiting Southwestern France, as well as the Northern and Western parts of Spain), and the Southeastern (SE; inhabiting the Danube Delta in Romania), as ascertained by Michaux et al. [28] and Cabria et al., [30]. To avoid a pseudoreplication problem, the results reported by the abovementioned authors were screened for possible haplotype duplications and, thus, data redundancy. As a result, some sequence overlaps were detected, as indicated in Table S6. However, they were assessed as negligible due to the fact that unique haplotypes, not individuals, were analyzed.

For each population, the number of detected haplotypes was determined (Table 1 and Tables S1–S5). The value of haplotype differentiation within each of the studied populations was determined, and the significance of the identified differences was tested. For this purpose, the method of comparing the Shannon–Wiener index [31] values developed by Zar [32] and based on the Hutcheson t-test was used. The analysis was performed using the sample-size-based rarefaction and extrapolation (R/E) sampling curve. This curve plots diversity estimates with confidence intervals as a function of the sample size up to double the reference sample size [33]. By the extrapolation of the estimated rarefaction curves, we estimated the additional number of sampled individuals needed to detect the total estimated haplotype richness in the general population.

The sample completeness curves were also drawn to determine the haplotype richness given the current sample sizes and then to determine how much the haplotype richness increased if the entire population was sampled. Additionally, the jackknife estimate of the haplotype richness [12] was calculated for each population, as well as the extrapolated values of the Shannon–Wiener index being determined, assuming full sample coverage. These analyses were performed using the R package iNEXT for the interpolation (rarefaction) and extrapolation of the Hill numbers, representing an intuitive and statistically rigorous diversity measure [33,34,35].

## 3. Results

The largest amount of intra-population genetic diversity, expressed by the number of identified haplotypes, was found in the NE population. It was five times higher than that in the SE population and as much as ten times higher than that in the W population (Table 2). For the SE population, the sample size was more than half the number of the examined individuals from the NE population; the W population was represented by a sample almost 25% larger than that for the NE population.

The comparison of the Shannon–Wiener index values for haplotype diversity confirms the observed relationships for the level of inter-population differentiation, expressed by the numbers of haplotypes identified in individual populations. Statistically significant differences were identified, with the *p*-values much lower than 0.01% (Table 2).

**Figure 1:**
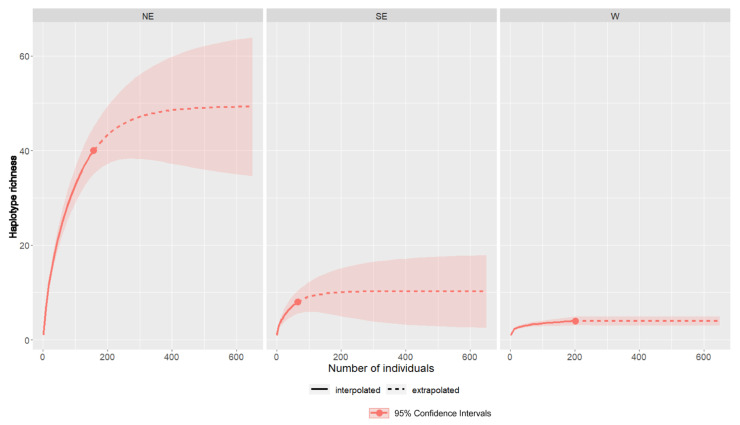
Sample-size-based refraction/extrapolation curves for three preserved populations (population abbreviations are as described in the main text) of European mink, plotting diversity estimates with confidence intervals as a function of sample size up to double the reference sample size (red dot indicates the interpolated haplotype richness, with the genetic data resources studied to date).

The R/E curves, determined on the basis of the research into the intraspecific genetic diversity of *M. lutreola* to date, are presented in Figure 1. They present the current (interpolated) dynamics of the increase in the number of detected haplotypes depending on the sample size. They also illustrate the predicted (extrapolated) increase in the number of haplotypes detected as the sample size increases above the current level. The greatest potential for an increase in the number of detected haplotypes (to a level of around 50) characterizes the NE population. On the other hand, an analogous analysis of the results of previous studies of the W population, despite the largest sample size, showed the lowest growth dynamic for the current known haplotype richness and its suppression at the level of four haplotypes in the case of a potential sample increase above the current state. In the case of the SE population, the R/E curve shows the possibility of increasing the number of haplotypes detected to the level of about 10, with the sample size exceeding the 200 examined individuals.

**Figure 2:**
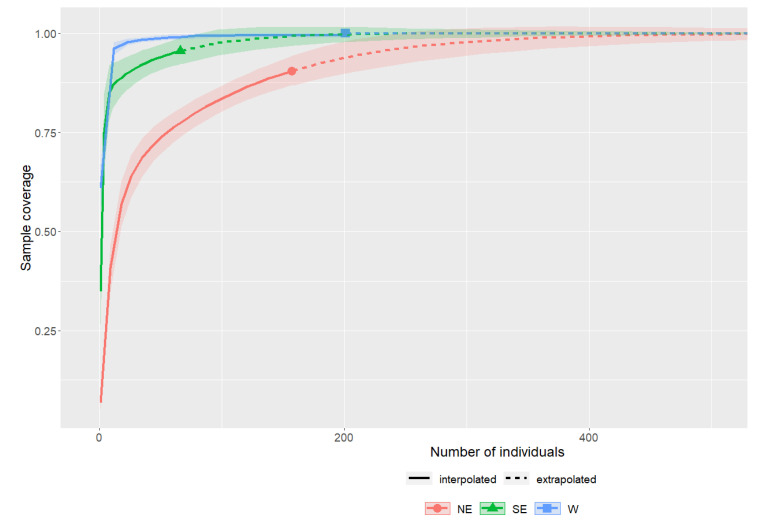
The sample completeness curves for three preserved populations of European mink (1 on the vertical axis means reaching the level of full coverage of the predicted, real level of haplotype richness of the general population; population abbreviations are as described in the main text).

The sample coverage, defined as the total probability of the occurrence of the mtDNA haplotype observed in the sample, predicted for the analyzed studies is presented in Figure 2. Given the current number of samples from the NE population (i.e., 157 individuals), it is estimated that we have achieved 87% sample coverage. To achieve 100% sample coverage, the sample size would have to more than double. A similar situation occurs for the SE population, with the sample coverage estimated at 96%; however, doubling the sample size compared to the current state would bring a much smaller gain in knowledge. In turn, any increase in the number of examined individuals from the W population is not expected to bring a significant increase in the number of haplotypes detected above the level determined by previous research (Figure 2).

The results of the analysis of how full (predicted) sample completeness would change the values of the Shannon–Wiener index for the haplotype richness of the studied populations, compared to the current one, are presented in Table 3. Increasing the sample size to about 400 individuals would cause a threefold increase in the level of haplotype richness, measured by the value of the Shannon–Wiener index, in the case of the W and SE populations, while in the case of the NE population, the increase would be more than eightfold. Different in value, but similar in terms of the proportion of differences between individual populations, are the jackknife estimates of the haplotype richness (95% confidence intervals indicated), equal to 55.50 ± 7.84, 14.00 ± 3.18 and 4.75 ± 0.87 for the NE, SE and W populations, respectively.

## 4. Discussion

The effects of sample size on population genetic diversity estimates have been investigated for different species (e.g., [4,9,11,18,19,20]). The rationalization of the sampling effort is particularly important for research in the field of the population genetics of species of high conservation concern, such as European mink. The fact that only about 5000 individuals persist in the wild significantly limits the availability of research material, but at the same time, the survival and restoration of the species depends on the implementation of evidence-informed conservation strategies [3,36]. For these reasons, and also due to the relatively low research interest in *M. lutreola* genetics, a highly efficient approach to the results of previous population genetic studies, in terms of obtaining the full meta-analytical benefit from them, is very important [3].

The conclusions drawn from the conducted meta-analysis confirm the statement that the NE and W populations of European mink exhibit the highest and the lowest genetic diversity, respectively, while the SE population is characterized by intermediate genetic diversity. Thus, they support the claim of very limited gene flow between the scattered study populations from three distinct groups of natural local populations [28,30]. It should be emphasized that the conducted meta-analysis covered studies from 2000 to 2015, and with the observed continuous decline of wild populations of European mink, a simultaneous depletion of the gene pool (number of haplotypes) should be expected. The potential haplotype variation of the general population was thus the greatest in the earliest study by Davison et al. [26].

Our results illustrate the size of the gap between measured and putative inter-population genetic diversity, reported in previous population genetic studies on European mink. The potential to detect haplotypes that have not been previously captured (and, thus, to more accurately measure the resources of genetic variability) is the greatest for the NE population, which is, in fact, a group of local populations inhabiting the Volga and Dvina drainage basins in the European parts of Russia, Belarus (extinct) and Estonia (extinct) [24,28,30]. This indicates the urgent need for conservation measures for the preserved Russian populations, both for maintaining the richness of the natural gene pool and due to the possibility of supplying the captive breeding stock with individuals from this area, with a view for future reintroductions [3,28,37]. This postulate takes on special significance in the context of the critical situation of the species in Russia, evidenced by the fact that *M. lutreola* remains on the list of game species in the country and only the Caucasian subspecies, *M. lutreola turovi*, is legally protected [24,38]. Additionally, a rapid decline and genetic depauperation of the extant local populations in the country are observed [24,29].

The obtained results demonstrate a simple and efficient predictive method for identifying the extent of uncaptured genetic diversity, defined by the number of haplotypes in the population and haplotype frequencies. This method allows determining the accuracy of genetic diversity tests, where the accuracy is defined as the difference between an estimate and the true value of a genetic differentiation indicator [39]. The conducted analysis can be treated both as a large-scale pilot study, informing sampling schemes for future population genetic studies, and an ex post assessment, supporting the planning of conservation measures. The method used in the present study allows us to apply the so-called stopping rule, an indication of the point beyond which further sampling does not increase the sampling coverage and, thus, is fruitless [2].

The validity of using a method based on the haplotype richness to estimate the completeness of the haplotype sampling, by analogy to the species richness, commonly applied in biodiversity studies, has been previously proven [2]. Additionally, the Shannon–Wiener index, originally adopted as a measure of the biodiversity, is widely used in population genetic studies. It should be pointed out that its use to measure genetic diversity is sometimes questioned as being negatively biased at small sample sizes [15]. However, due to the comparative nature of the analyses in this work, the possible influence of the sample size on the result basically confirms our interpretation, additionally pointing to the advisability of more detailed studies of the SE population (as indicated by the results of the jackknife analysis).

## 5. Conclusions

Taken together, our results have several implications. Firstly, they demonstrate the practical utility of the method with respect to informing sampling design and accounting for sampling uncertainty in population genetic studies. As appropriate statistical advice should be sought at the research planning stage to ensure that the designed sampling scheme will enable the research questions to be properly and accurately answered, our results have implications for the design of future population genetic research on European mink. Specifically, further research on the NE population, with an increased sample size, has the greatest potential to detect new haplotypes and, thus, diagnose the actual intra-population genetic diversity, while this potential in the case of the SE population is low or moderately low, and increasing the sample size for the W population is expected not to generate new knowledge. Secondly, taking into account the serious challenges in obtaining large samples of individuals, due to *M. lutreola* being a rare and difficult-to-capture species of high conservation concern [24,36], the results we report are especially relevant for correcting assumptions based on the results of the research by Davison et al. [26], Michaux et al. [27,28], Korablev et al., [29] and Cabria et al. [30], according to which conservation priorities are set. This approach stems from the belief that the results of previous research should be fully exploited, in accordance with a rational approach to the use of biological material, as well as the financial outlays, time and effort invested in research. It is worth mentioning that, for non-model species, such as European mink, large numbers of genetic markers (e.g., microsatellite loci and mtDNA haplotypes) are often not available, so every study in this field is important and highly valued [3,18]. Thirdly, the relatively low sample coverage for the NE population that we have shown in this study, in connection with the above-described situation of the species in Russia and great importance of the local populations for conservation and restoration efforts, clearly indicates the urgent need to take immediate conservation measures for European mink in this country.

Questions about the effects of the number of markers, type of marker applied and possible multiple sampling on the detection of the real genetic diversity of wild populations of European mink have not been addressed in the present study and need to be further investigated. Similarly, the effect that different sample sizes will have on low-coverage genomic resequencing or reduced-representation sequencing, such as genotyping by sequencing (GBS) or restriction-site-associated DNA sequencing (RAD-Seq), needs to be addressed. Our results imply that, while moderate coverage for all populations is needed, a larger sample for the NE population should be recommended because, with a higher genetic diversity, more minor alleles are expected, which could be lost with filtering, resulting in an underestimate of the genetic diversity present.

## Figures and Tables

**Table 1:** Genetic diversity measures reported in the studies used in the meta-analysis performed in the present study (population abbreviations are as described in the main text).

Author	Population	Number of Individuals		Number of Haplotypes		*π*	SD	*h*	SD
[27]	NE	14	43	11	14	0.0197	0.0025	0.9780	0.0350
	SE	2		2		0.0039	0.0019	1.0000	0
	W	27		1		0	0	0	0
[28]	NE	18	176	10	15	0.0120	0.0014	0.9390	0.0580
	SE	34		4		0.0012	0.0003	0.4690	0.0880
	W	124		1		0	0	0	0
[29]	NE	11	11	8	8	0.0092	0.0055	0.9500	0.0540
[26]	NE	30	37	4	6	0.0008	0.0002	0.2460	0.0720
	W	7		2		0.0024	0.0012	0.2640	0.1360
[30]	NE	84	157	13	18	0.0040	0.0030	0.8620	0.0160
	SE	30		4		0.0019	0.0015	0.3520	0.0103
	W	43		1		0	0	0	0

NE—Northeastern population; SE—Southeastern population; W—Southwestern population; *π*—nucleotide diversity [21]; *h*—haplotype diversity [21]; SD—standard deviation.

**Table 2:** Comparison of the haplotype richness of three remaining populations of European mink.

Population	NE	SE	W
Number of individuals	157	66	201
Number of haplotypes (haplotype richness)	40	8	4
Shannon–Wiener index	3.068	1.295	0.676
Standard deviation of Shannon–Wiener index	0.09252	0.12501	0.05593
Degrees of freedom	140	140	202
Level of statistical significance of difference between Shannon–Wiener index values	0.000 *	0.000 *	0.000 *
Direction of comparison	NE to W	SE to NE	W to SE

The number of haplotypes (haplotype richness) is the sum of the unique haplotypes found by different authors for a particular population. Asterisk (*) indicates statistical significance. Population abbreviations are as described in the main text.

**Table 3:** Predicted values of the Shannon–Wiener index for haplotype richness of the preserved populations of *Mustela lutreola*, assuming full sample representativeness (population abbreviations as described in the main text).

Population	Extrapolated Shannon–Wiener Index	Standard Error	Lower 95% Confidence Limit	Upper 95% Confidence Limit
NE	25.648	2.272	21.493	30.102
SE	3.956	0.499	3.651	4.934
W	1.982	0.115	1.966	2.207

## Data Availability

The data presented in this study are available in the Supplementary Materials.

## Supplementary Materials

The following are available online at https://www.mdpi.com/article/10.3390/genes12101555/s1, Table S1: mtDNA haplotypes identified in population genetic studies of Davison et al. [26], used in the meta-analysis conducted in the present paper (population abbreviations as described in the main text), Table S2: mtDNA haplotypes identified in population genetic studies of Michaux et al. [27], used in the meta-analysis conducted in the present paper (population abbreviations as described in the main text), Table S3: mtDNA haplotypes identified in population genetic studies of Michaux et al. [28], used in the meta-analysis conducted in the present paper (population abbreviations as described in the main text), Table S4: mtDNA haplotypes identified in population genetic studies of Korablev et al. [29], used in the meta-analysis conducted in the present paper (population abbreviations as described in the main text), Table S5: mtDNA haplotypes identified in population genetic studies of Cabria et al. [30], used in the meta-analysis conducted in the present paper (population abbreviations as described in the main text), Table S6: List of haplotypes identified in previous studies on population genetics of European mink and used to conduct the current meta-analysis.
