# Supplementary material for: A Meta-Analytical Investigation of the Gap between Measured and Predicted Inter-Population Genetic Diversity in Species of High Conservation Concern—The Case of the Critically Endangered European Mink Mustela lutreola L., 1761
Source: Genes (Basel). 2021 Sep 29;12(10):1555. doi: 10.3390/genes12101555 (PMC8535868; doi:10.3390/genes12101555)
Supplement: Supplementary file 1 [file genes-12-01555-s001.zip › genes-1366668-supplementary.pdf]

genes

MDPI

Supplementary Materials

Table S1. mtDNA haplotypes identified in population genetic studies of Davison et al. [26], used in the meta-analysis conducted in the present paper (population abbreviations as described in the main text)

Population	Haplotype					N	
	C5	C11	C17	C18	C19		
NE	0	26	2	1	1	30	37
W	1	6	0	0	0	7	

Table S2. mtDNA haplotypes identified in population genetic studies of Michaux et al. [27], used in the meta-analysis conducted in the present paper (population abbreviations as described in the main text)

Population	Haplotype																	N	
	DL1	DL2	DL3	DL4	DL5	DL6	DL7	DL8	DL9	DL10	DL11	DL12	AF207720	AF207721	AF207722	AF207723	AF207724		
NE	1	1	1	1	1	1	0	0	0	1	1	1	1	1	1	1	1	14	43
SE	0	0	0	0	0	0	1	1	0	0	0	0	0	0	0	0	0	2	
W	0	0	0	0	0	0	0	0	27	0	0	0	0	0	0	0	0	27	

Table S3. mtDNA haplotypes identified in population genetic studies of Michaux et al. [28], used in the meta-analysis conducted in the present paper (population abbreviations as described in the main text)

Population	Haplotype															N	
	DL1	DL2	DL3	DL4	DL5	DL6	DL7	DL8	DL9	DL10	DL11	DL12	DL19	DL20	DL21		
NE	1	1	2	1	1	1	0	0	0	3	3	2	0	0	3	18	176
SE	0	0	0	0	0	0	1	30	0	0	0	0	1	2	0	34	
W	0	0	0	0	0	0	0	0	124	0	0	0	0	0	0	124	

Table S4. mtDNA haplotypes identified in population genetic studies of Korablev et al. [29], used in the meta-analysis conducted in the present paper (population abbreviations as described in the main text)

Population	Haplotype								N
	dl28t	dl19t	dl30t	dl18t	dl14o	dl32n	dl107o	dl6n	
NE	2	1	1	2	1	1	1	2	11

Table S5. mtDNA haplotypes identified in population genetic studies of Cabria et al. [30], used in the meta-analysis conducted in the present paper (population abbreviations as described in the main text)

Population	Haplotype																	N	
	Mlh1	Mlh2	Mlh3	Mlh4	Mlh5	Mlh6	Mlh7	Mlh8	Mlh9	Mlh10	Mlh11	Mlh12	Mlh13	Mlh14	Mlh15	Mlh16	Mlh17		
NE	13	2	2	15	1	2	3	0	0	0	0	3	11	10	1	20	1	84	157
SE	0	0	0	0	0	0	0	24	1	1	0	0	0	0	0	4	0	30	
W	0	0	0	0	0	0	0	0	0	0	43	0	0	0	0	0	0	43	

Table S6. List of haplotypes identified in previous studies on population genetics of European mink and used to conduct the current meta-analysis

Haplotype (names according to the publications cited)	GenBank accession number	bp	Mitochondrial genome section	Position relative to the reference sequence MT304869 (bp)
Davison et al. 2000				
C5	AF068538	337	*Cytb*	14.205-14.541
C11	AF068544	337	*Cytb*	14.205-14.541
C17	AF207712	337	*Cytb*	14.205-14.541
C18	AF207713	337	*Cytb*	14.205-14.541
C19	AF207714	337	*Cytb*	14.205-14.541
Michaux et al. 2004 & 2005				
DL1	AJ548803	730	D-loop	15.470-16.040 […] 16.240-16.411
DL2	AJ548804	727	D-loop	15.471-16.040 […] 16.240-16.411
DL3	AJ548805	730	D-loop	15.470-16.040 […] 16.240-16.411
DL4	AJ548806	720	D-loop	15.471-16.038 […] 16.249-16.408
DL5	AJ548807	564	D-loop	15.471-16.036
DL6	AJ548808	728	D-loop	15.471-16.040 […] 16.240-16.411
DL7	AJ548809	729	D-loop	15.471-16.040 […] 16.240-16.411
DL8	AJ548810	727	D-loop	15.471-16.040 […] 16.240-16.411
DL9	AJ548811	730	D-loop	15.470-16.040 […] 16.240-16.411
DL10	AJ548812	727	D-loop	15.471-16.040 […] 16.240-16.411
DL11	AJ548813	730	D-loop	15.470-16.040 […] 16.240-16.411

DL12	AJ548814	617	D-loop	15.471-16.040 […] 16.240-16.411
DL19	Michaux 2005 (pers. com.)	731	D-loop	15.470-16.040 […] 16.240-16.411
DL20	Michaux 2005 (pers. com.)	571	D-loop	15.470-16.040
DL21	Michaux 2005 (pers. com.)	566	D-loop	15.470-16.036
D18	AF207720	365	D-loop	15.360-15.725
D19	AF207721	366	D-loop	15.360-15.725
D20	AF207722	365	D-loop	15.360-15.725
D21	AF207723	365	D-loop	15.360-15.725
D22	AF207724	366	D-loop	15.360-15.725
Korablev et al. 2014				
dl28t	JX982497	525	D-loop	15.470-15.995
dl19t	JX982501	525	D-loop	15.470-15.995
dl30t	JX982499	256	D-loop	15.470-15.996
dl18t	JX982500	525	D-loop	15.470-15.995
dl114o	JX982502	525	D-loop	15.470-15.995
dl32n	JX982496	525	D-loop	15.470-15.995
dl107o	JX982495	525	D-loop	15.470-15.995
dl6n	JX982498	525	D-loop	15.470-15.995
Cabria et al. 2015				
Mlh1	EU548035	502	*tRNA-Thr*, *tRNA-Pro*, D-loop	15.260-15.762
Mlh2	EU548036	502	*tRNA-Thr*, *tRNA-Pro*, D-loop	15.260-15.762
Mlh3	EU548037	502	*tRNA-Thr*, *tRNA-Pro*, D-loop	15.260-15.762
Mlh4	EU548038	502	*tRNA-Thr*, *tRNA-Pro*, D-loop	15.260-15.762
Mlh5	EU548039	503	*tRNA-Thr*, *tRNA-Pro*, D-loop	15.260-15.762
Mlh6	EU548040	503	*tRNA-Thr*, *tRNA-Pro*, D-loop	15.260-15.762
Mlh7	EU548041	503	*tRNA-Thr*, *tRNA-Pro*, D-loop	15.260-15.762
Mlh8	EU548042	501	*tRNA-Thr*, *tRNA-Pro*, D-loop	15.260-15.762
Mlh9	EU548043	501	*tRNA-Thr*, *tRNA-Pro*, D-loop	15.260-15.762
Mlh10	EU548044	501	*tRNA-Thr*, *tRNA-Pro*, D-loop	15.260-15.762
Mlh11	EU548045	503	*tRNA-Thr*, *tRNA-Pro*, D-loop	15.260-15.762
Mlh12	EU548046	503	*tRNA-Thr*, *tRNA-Pro*, D-loop	15.260-15.762
Mlh13	EU548047	502	*tRNA-Thr*, *tRNA-Pro*, D-loop	15.260-15.762
Mlh14	EU548048	502	*tRNA-Thr*, *tRNA-Pro*, D-loop	15.260-15.762
Mlh15	EU548049	502	*tRNA-Thr*, *tRNA-Pro*, D-loop	15.260-15.762
Mlh16	EU548050	502	*tRNA-Thr*, *tRNA-Pro*, D-loop	15.260-15.762
Mlh17	EU548051	501	*tRNA-Thr*, *tRNA-Pro*, D-loop	15.260-15.762
